# The Conduct of Ordinary Labor through External Examinations Solely

**Published:** 1894-09

**Authors:** 


### THE CONDUCT OF ORDINARY LABOR THROUGH EXTERNAL EXAMINATIONS SOLELY.

Drs. Leopold and Spörlin make a warm plea for limiting examinations made in the course of ordinary labor, to the external parts, and adduce the advantages of such a course (*Med. News*). Infection is thereby avoided; the natural sense of modesty of the parturient is not offended; and careless rupture of the membranes is avoided. Skill in external examination is acquired with reasonable readiness. In the large majority of cases such examination alone is sufficient for the recognition of the position and presentation of the fetus, and for the study of the course of an ordinary labor. As there can be no objection to its frequent exercise, abnormalities of parturition may the more readily be detected early, and means of correction promptly employed. Experience soon teaches the difference in the position of the fetus assumed in case of pelvic contraction on the part of the mother. The position and presentation having been recognized by external examination, internal examination for the determination of possible pathologic conditions of the birth canal need be but brief, and can be conducted with great care. For the attainment of this desirable result it is essential that the obstetric pupil familiarize himself thoroughly with the conditions of normal labor as determined by physical external examinations, as well as with the physiology of normal labor. Obstetric operations are principally to be taught upon the phantom.—*St. Louis Med. and Surg. Journal.*

